# A prospective cohort study comparing the reactogenicity of trivalent influenza vaccine in pregnant and non-pregnant women

**DOI:** 10.1186/s12884-015-0495-2

**Published:** 2015-03-18

**Authors:** Annette K Regan, Lauren Tracey, Christopher C Blyth, Donna B Mak, Peter C Richmond, Geoffrey Shellam, Caroline Talbot, Paul V Effler

**Affiliations:** School or Pathology and Laboratory Medicine, University of Western Australia, 227 Stubbs Terrace Shenton Park, Western Australia, Australia; Communicable Disease Control Directorate, Western Australia Department of Health, Shenton Park, Western Australia, Australia; School of Paediatrics and Child Health, University of Western Australia, Crawley, Western Australia Australia; Vaccine Trials Group, Telethon Kids Institute, University of Western Australia, Subiaco, Western Australia Australia

**Keywords:** Trivalent influenza vaccine, Pregnancy, Vaccine safety, Antenatal immunisation

## Abstract

**Background:**

Influenza vaccination during pregnancy can prevent serious illness in expectant mothers and provide protection to newborns; however, historically uptake has been limited due to a number of factors, including safety concerns. Symptomatic complaints are common during pregnancy and may be mistakenly associated with reactions to trivalent influenza vaccine (TIV). To investigate this, we compared post-vaccination events self-reported by pregnant women to events reported by non-pregnant women receiving TIV.

**Methods:**

A prospective cohort of 1,086 pregnant women and 314 non-pregnant female healthcare workers (HCWs) who received TIV between March-May 2014 were followed-up seven days post-vaccination to assess local and systemic adverse events following immunisation (AEFIs). Women were surveyed by text message regarding perceived reactions to TIV. Those reporting an AEFI completed an interview by telephone or mobile phone to ascertain details. Logistic regression models adjusting for age and residence were used to compare reactions reported by pregnant women and non-pregnant HCWs.

**Results:**

Similar proportions of pregnant women and non-pregnant, female HCWs reported ≥1 reaction following vaccination with TIV (13.0% and 17.3%, respectively; OR = 1.2 [95% CI: 0.8-1.8]). Non-pregnant, female HCWs were more likely to report fever or headache compared to pregnant women (OR: 4.6 [95% CI 2.1-10.3] and OR: 2.2 [95% CI 1.0-4.6], respectively). No other significant differences in reported symptoms were observed. No serious vaccine-associated adverse events were reported, and less than 2% of each group sought medical advice for a reaction.

**Conclusions:**

We found no evidence suggesting pregnant women are more likely to report adverse events following influenza vaccination when compared to non-pregnant female HCWs of similar age, and in some cases, pregnant women reported significantly fewer adverse events. These results further support the safety of TIV administered in pregnant women.

## Background

The World Health Organisation has identified pregnant women as the highest priority for influenza vaccination [1]. Despite national recommendations in Australia and the availability of free vaccine under the National Immunisation Program, surveys have found that less than 30% of pregnant women in Australia are immunised against seasonal influenza [2,3]. A number of studies have confirmed influenza antenatal vaccination is safe for mother and baby [4-10]. However, continued monitoring is warranted, considering the antigenic composition can vary from year to year and ongoing concerns about side-effects remain a common factor contributing to non-vaccination among antenatal patients [2,11-13]. Even the expectation of minor post-vaccination reactions can negatively affect the decision to be immunised against influenza [14,15]. Pregnancy can be associated with a variety of symptomatic complaints and whether these impact the side effects reported by antenatal influenza vaccine recipients is currently unknown. To assess this, we compared post-vaccination reactions among pregnant women to those reported by non-pregnant females of similar age in Western Australia.

## Methods

In 2012, the Western Australia Department of Health (WA-DOH) initiated a program for active surveillance of adverse events following immunisation (AEFI) in pregnant women. The Follow-up and Active Surveillance of Trivalent influenza vaccine in Mums (FASTMum) program follows up a subset of pregnant women who receive trivalent influenza vaccine (TIV), beginning in March each year. Antenatal women receiving government-procured TIV are asked by their provider at the time of immunisation if they are willing to be contacted by the WA-DOH for quality assurance purposes. In 2014, the opportunity for post-vaccination follow-up was extended to healthcare workers (HCWs) immunised against influenza at government hospitals and health centres.

Using an automated system, pregnant women and HCWs consenting to follow up were sent a short message service (SMS) seven days after they had been vaccinated with TIV. The SMS read:

“This is a message from the WA Department of Health. Our records show that you recently had a flu vaccine and we are conducting routine follow up. Please respond Y if you experienced any kind of reaction, fever, or illness in the week following your vaccination, or N if there was no reaction”.

Persons who replied “Y”, “yes” or some other affirmative response by SMS were sent a follow-up message soliciting details regarding the possible AEFI they reported experiencing. The second message read:

“Thank you, your ongoing health is important to us. Please click here to answer a five minute survey about your reaction. Alternatively, please respond CALL if you would prefer to be telephoned about your reaction”.

The second SMS included an embedded link to a survey which could be completed on a mobile phone. Research nurses subsequently attempted to telephone and interview anyone who had not responded to either the first or second SMS, or had not completed the mobile phone survey, as well as those who had replied “Call” by SMS.

For this analysis, all non-pregnant, female healthcare workers (HCWs) in the follow-up program, who were of reproductive age and were vaccinated with the same brand of TIV, i.e. Vaxigrip® (Sanofi Pasteur) were selected for comparison with pregnant women. Female HCWs were eligible for the analysis if they were between the ages of 18 and 45 years and had indicated on their consent form that they were not pregnant at the time of vaccination. The majority (82%) of pregnant women included in the analysis were in their second or third trimester of pregnancy; 93% of reported vaccinations in pregnant women and non-pregnant female HCWs were included in the follow-up. Participants who provided no telephone number (5%), provided only a home telephone number (2%) or an incorrect mobile telephone number (<1%) on their consent form were excluded. Ethics approval for this assessment was obtained by the University of Western Australia Human Research Ethics Committee (RA/4/1/6095).

### Survey instrument

The mobile phone and telephone questionnaires asked if the vaccinee had experienced fever, headache, fatigue, rigors, convulsions, vomiting, or pain or swelling at the injection site - indicated by a “yes” or “no” response to each. The presence or absence of other symptoms was solicited and, if present, recorded verbatim. Respondents were also asked to recall the time between vaccination and first symptom onset as well as the duration of any symptoms reported. Consumption of over-the-counter antipyretic or pain relievers following the vaccination was queried, as well as whether the vaccinee had called a general practitioner (GP) or other health service for telephone medical advice regarding the reaction, or had visited a GP, after-hours clinic, or emergency department (ED) to receive treatment for a reported reaction.

### Outcome measurement

The occurrence of any AEFI was defined as a “yes” response to the initial SMS message. A systemic reaction was defined as a “yes” response to fever, headache, fatigue, vomiting, rigors, or self-reported cold and flu-like symptoms, myalgia, nausea, or malaise. A local reaction was defined as replying “yes” to pain or swelling at the injection, or self-reported redness at the site of injection. A reaction requiring telephone advice was defined as any AEFI where the woman reported calling a GP, a nurse helpline, or other healthcare service for advice regarding their reaction. A reaction requiring medical attention was defined as any AEFI where the woman reported visiting a GP or other health service for the reaction. An AEFI requiring treatment included any AEFI which was self-treated with an anti-pyretic/analgesic following vaccination and those receiving treatment by a medical professional.

### Statistical analysis

Statistical analysis was performed using SAS version 9.3 (SAS Institute, North Carolina, United States). Overall response rate was calculated based on the proportion of women who replied by either SMS or telephone and provided complete details regarding any adverse events out of all women contacted. Initial comparisons between AEFI reported by pregnant women and non-pregnant, female HCWs were made using Fisher’s exact test. Adjusted analyses controlling for demographic differences observed between groups were performed using multivariate logistic regression models. Differences in the mean symptom onset and symptom duration were compared with independent sample t-tests using the Satterthwaite approximation for degrees of freedom. A power analysis indicated the acquired sample size was sufficient to determine differences between groups at a power level of 0.98.

## Results

**Figure 1:**
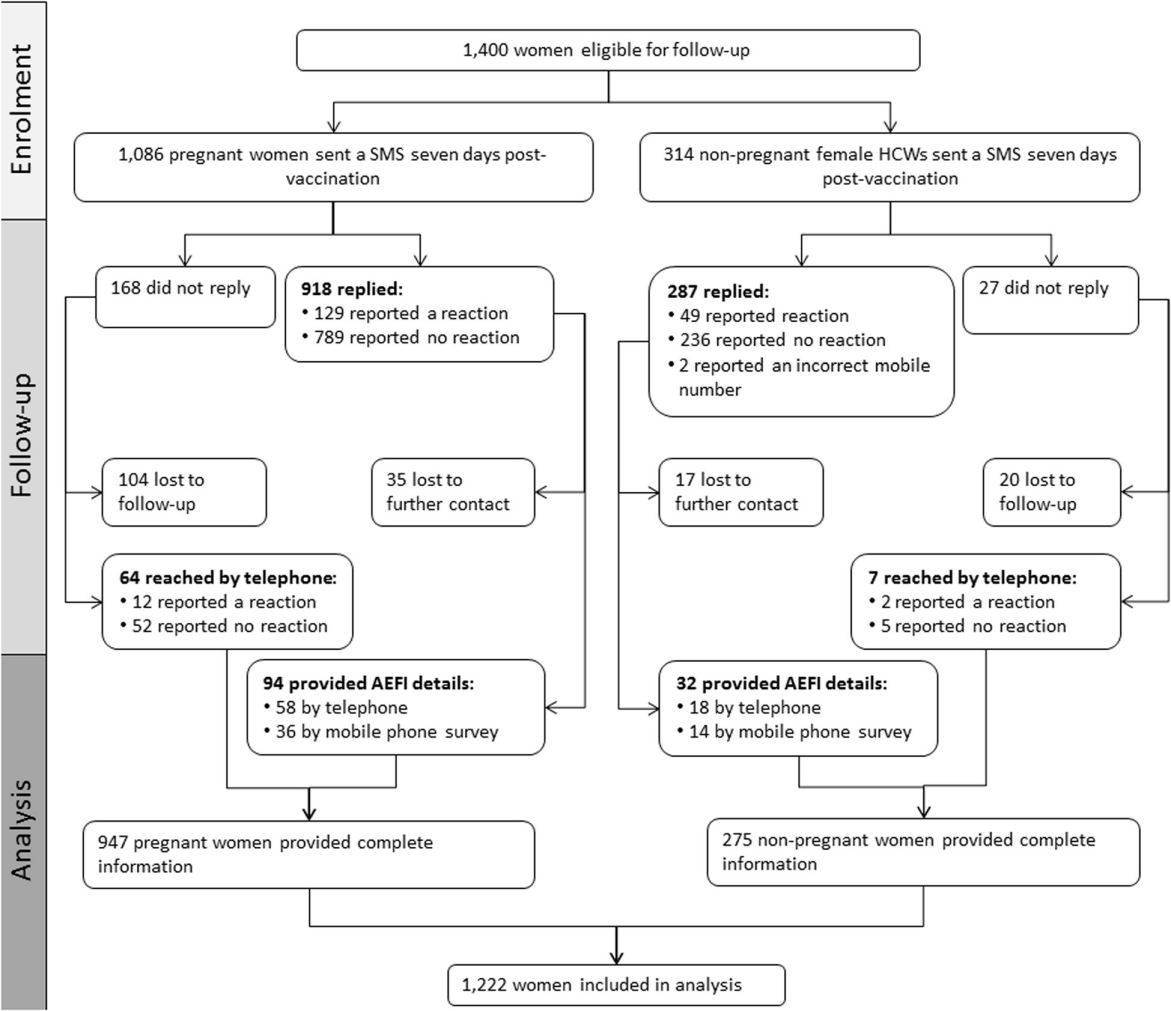
**Follow-up of adverse events following trivalent influenza vaccine in pregnant women and non-pregnant female healthcare workers – FASTMum, Western Australia, Australia, 19 March- 15 May 2014.**

Between 19 March and 15 May 2014, a total of 1,400 women (1,086 pregnant and 314 non-pregnant HCW) were sent the SMS asking about possible AEFI (Figure 1); 1,205 (86%) women replied by SMS (918 [85%] pregnant and 287 [91%] non-pregnant), and another 71 (64 [6%] pregnant and 7 [2%] non-pregnant) did not reply but were surveyed later by telephone. A total of 52 women (35 [4%] pregnant and 17 [6%] non-pregnant) who replied to the initial SMS and indicated they had experienced a reaction did not provide AEFI details and were excluded from analysis. The final analysis included 947 pregnant women and 275 non-pregnant women. The overall response rates in pregnant women (87.2% [95% CI 85.2-89.2%]) and non-pregnant, female HCWs (87.6% [95% CI 83.9-91.2%]) were similar (p > .05).

Two significant differences in demographic characteristics were observed between the cohorts of pregnant women and non-pregnant, female HCWs included in our study. Non-pregnant, female HCWs were on average 2.6 years older than our cohort of pregnant women (33.7 years vs. 31.1 years, respectively, *p* < .01), and were also more likely to reside in a non-urban area (47.8% [95% CI 42.2-53.3%]) compared to pregnant women (15.6% [95% CI 13.3-17.9%]; *p* < .01). The greater proportion of non-pregnant female HCWs residing in non-urban areas is likely because many metropolitan health care facilities offered HCWs an intra-dermal influenza vaccine in preference to Vaxigrip® (Sanofi Pasteur).

**Figure 2:**
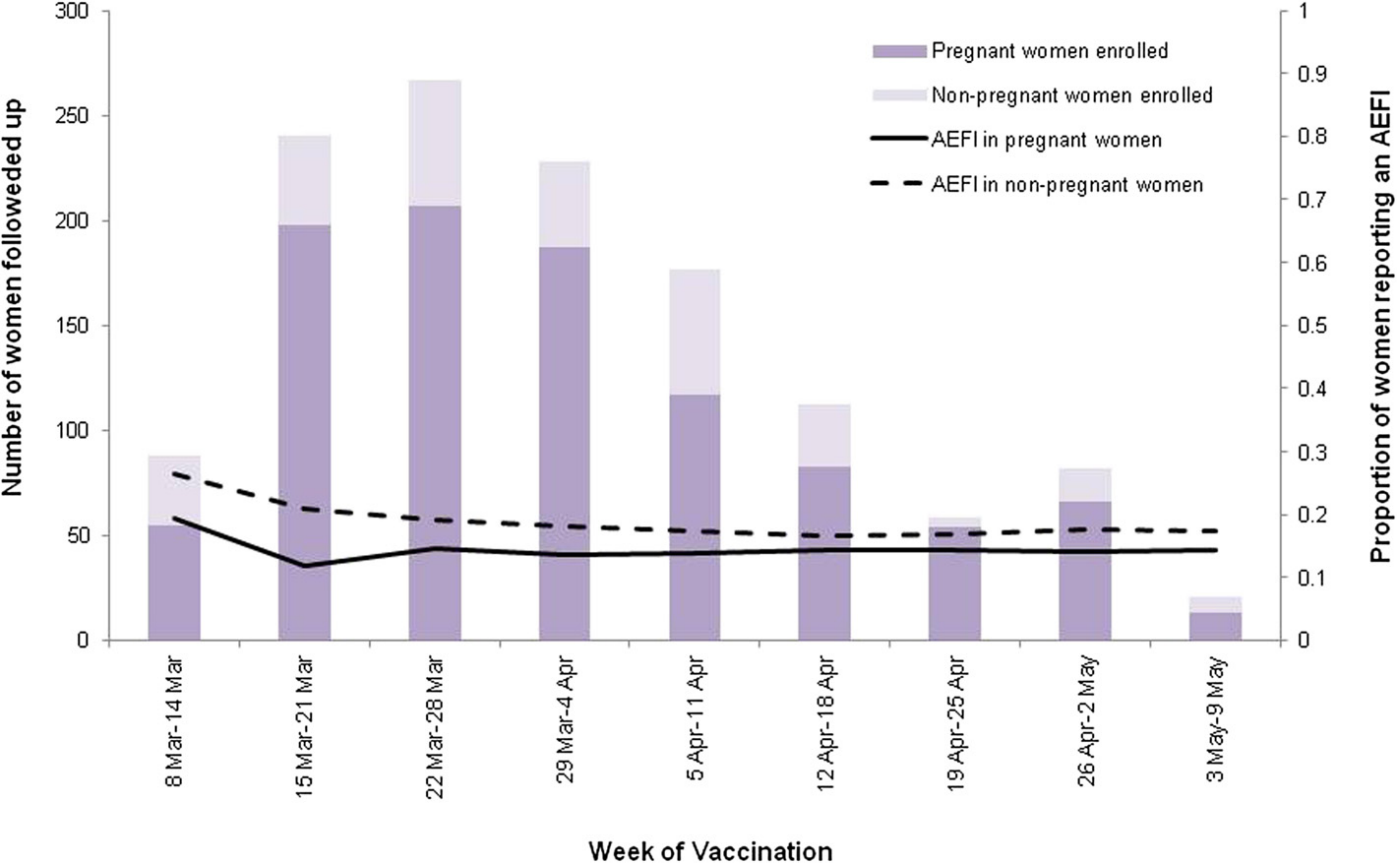
**Proportion of pregnant and non-pregnant women reporting an adverse event following trivalent influenza vaccination – FASTMum, Western Australia, Australia, 19 March- 15 May 2014.**

A total of 192 (15.7%) women reported a suspected reaction, with similar proportions of pregnant and non-pregnant, female HCWs reporting at least one AEFI (13.0% [95% CI 11.0-15.0%] and 17.3% [95% CI 13.0-21.6%], respectively; *p* = .34) (Table 1). The rate of reaction was constant for both pregnant women and non-pregnant, female HCWs throughout the study period (Figure 2). The most common reaction reported by both pregnant and non-pregnant HCWs was a local reaction (4.5% [95% CI 3.4-6.1%] and 7.3% [95% CI 4.1-10.5%], respectively, *p* = .13). No serious vaccine-associated reactions were reported. Systemic reactions were reported by similar proportions of pregnant women and non-pregnant, female HCWs, overall (9.0% and 10.2% among pregnant women and non-pregnant HCWs, respectively). However, fever (OR 4.6 [95% CI 2.1-10.3]) and headache (OR 2.2 [95% CI 1.0-4.6]) were both reported more frequently by non-pregnant HCWs than pregnant women. Four of the 16 non-pregnant HCWs and five of the 46 pregnant women who reported a fever reported measuring their temperature.

**Table 1:** **Adverse events following influenza immunisation reported by pregnant and non-pregnant women – FASTMum, Western Australia, Australia, 19 March-15 May 2014**

	**Pregnant (n = 947)**		**Non-pregnant (n = 275)**		**Fisher’s exact test p-value**	**AOR p-value**
	**n**	**Percent (95% CI)**	**n**	**Percent (95% CI)**		
Any reaction**	141	13.0 (11.0-15.0)	51	17.5 (13.1-21.8)	.19	.33
**Systemic reaction**	85	9.0 (7.1-10.8)	28	10.2 (6.6-13.8)	.55	.36
Fever	15	1.6 (0.8-2.4)	16	5.8 (3.0-8.6)	<.01*	<.01*
Headache	27	2.9 (1.8-3.9)	13	4.7 (2.2-7.3)	<.01*	.04*
Fatigue	40	4.2 (2.9-5.5)	13	4.7 (2.2-7.3)	.74	.68
Vomiting	7	0.7 (0.2-1.3)	0	(0.0-0.7)	.36	.95
Rigors	5	0.5 (0.1-1.0)	2	0.7 (0.0-1.7)	.66	.89
Cold/flu-like	37	3.9 (2.7-5.1)	10	3.6 (1.4-5.9)	.50	.69
Myalgia	11	1.2 (0.5-1.8)	5	1.8 (0.2-3.4)	.37	.71
Nausea	8	0.8 (0.3-1.4)	1	0.4 (0.0-1.1)	.69	.59
Malaise	4	0.4 (0.0-0.8)	1	0.4 (0.0-1.1)	.69	.51
**Local reaction**	45	4.8 (3.4-6.1)	20	7.3 (4.2-10.4)	.13	.13
**Other reaction**	6	0.6 (0.1-1.1)	2	0.7 (0.0-1.7)	.57	.89

**Any reaction was defined as replying “yes” to the question “did you experience any fever, illness, or reaction following your vaccination?”.

*Significant at α = .05.

AOR, adjusted odds ratio – adjusted for age and residence (metropolitan/non-metropolitan).

CI, confidence interval.

On average, reported fever began within 24 hours of vaccination (median: 24 hours; IQR: 6–48 hours) and lasted between 8–120 hours (median: 27 hours; IQR: 12–48 hours). The time to onset and duration of fever were similar in pregnant women and non-pregnant, female HCWs (*p* = .52 and *p* = .14, respectively). Other reported systemic reactions usually occurred within 24 hours of vaccination (median: 24 hours; IQR: 6–48 hours) and lasted for a median of 48 hours (IQR: 24–72 hours). The onset and duration of these reactions did not differ between pregnant women and non-pregnant, female HCWs (*p* = .26 and *p* = .21, respectively). Local reactions typically began on the day of vaccination (median: 8 hours; IQR: 3–24 hours) and had a median duration of 48 hours (IQR: 24–72 hours). The onset and duration of local reactions did not differ between pregnant women and non-pregnant, female HCWs (*p* = .18 and *p* = .24, respectively).

Almost twice as many non-pregnant, female HCWs reported a reaction for which they obtained some form of treatment, such as self-treatment with an antipyretic or pain reliever or treatment by a doctor, medical centre or hospital emergency department (10.7% [95% CI 6.6-14.8%]) compared with pregnant women (5.5% [95% CI 4.0-7.0%]). However, this difference was not statistically significant (*p* = .06) (Table 2). This difference in proportion of reactions treated between pregnant and non-pregnant, female HCWs can be largely attributed to the increased rates of fever and headache reported by non-pregnant, female HCWs. Among women reporting any reaction, headache and fever were the only symptoms significantly associated with seeking some form of treatment (*p* = .03 and *p* < .01, respectively). Reactions requiring telephone advice or medical attention were uncommon in both pregnant women and non-pregnant, female HCWs (1.3% [95% CI 0.5-2.0%] vs 0.4% [95% CI 0.0-1.2%], *p* = .25).

**Table 2:** **Medical attendance of adverse events following influenza immunisation among pregnant and non-pregnant women – FASTMum, Western Australia, Australia, 19 March-15 May 2014**

	**Pregnant (n = 947)**		**Non-pregnant (n = 275)**		**Fisher’s exact test p-value**	**AOR p-value**
	**n**	**Percent (95%** **CI)**	**n**	**Percent (95%** **CI)**		
Reaction requiring any treatment**	52	5.5 (4.0-6.9)	26	9.5 (6.0-12.9)	.02	.06
**Reaction requiring telephone advice**	11	1.2 (0.5-1.8)	2	0.8 (0.0-2.0)	.74	.95
Telephoned a doctor	7	0.7 (0.2-1.3)	0	0.0 (0.0-0.1)	.36	.95
Telephoned other	4	0.4 (0.0-0.8)	2	0.8 (0.0-2.0)	.62	.78
**Reaction requiring medical attention**	12	1.3 (0.5-2.0)	1	0.4 (0.0-1.2)	.32	.18
Visited a doctor	8	0.8 (0.3-1.4)	1	0.4 (0.0-1.2)	.69	.45
Visited a hospital emergency department	4	0.4 (0.0-0.8)	0	(0.0-0.1)	.58	.96

**A reaction requiring treatment was defined as any reaction where the woman reported self-treating with an antipyretic or pain reliever or visiting a doctor, medical centre or hospital emergency department to seek treatment.

AOR, adjusted odds ratio – adjusted for age and residence (metropolitan/non-metropolitan).

CI, confidence interval.

Four pregnant women reported attending a hospital emergency department in the week following influenza vaccination. One woman reported fever and rigors, a second reported gastroenteritis, the third reported an upper respiratory tract infection, and the fourth woman reported nausea, dizziness, malaise and a miscarriage. Follow-up assessment by the physician caring for the woman who reported a miscarriage indicated the woman had a history of obstetric complications, including polycystic ovarian syndrome and multiple previous miscarriages. The physician reported the event was consistent with a spontaneous abortion and unlikely to be related to vaccination.

## Discussion

We used SMS to collect information on post-influenza vaccination events in a sample of pregnant and non-pregnant women, and found no evidence that pregnant women are more likely to experience a reaction following administration of the 2014 influenza vaccination when compared to non-pregnant, female HCWs of similar age. Using active surveillance, we found that 1-in-10 pregnant women experienced some sort of reaction, but fewer than 2% developed a fever. These results were similar for non-pregnant, female HCWs, although this group reported slightly higher rates of fever and headache. The most common side-effect reported by either group was a local reaction at the injection site, occurring in about one of every 15–20 women vaccinated. This information is useful in reassuring pregnant women and antenatal immunisation providers regarding the reactogencity of seasonal influenza vaccination during pregnancy. However, because the antigenic characteristics of the influenza vaccine can change from year to year, ongoing assessments of safety and reactogencity are warranted. Secondarily, these results indicate that SMS is a feasible method of rapidly collecting data for monitoring vaccine safety in both pregnant and non-pregnant women.

Previous active surveillance initiatives in Western Australia in 2012 [9] and 2013 [10] found AEFI rates similar to those reported here for the 2014 influenza vaccine. Comparable rates of AEFI among pregnant women have been reported from other settings [10,16]. In the United States, Nordin et al. [16] investigated the incidence of medically-attended events in pregnant women 42 days following TIV vaccination, finding a low frequency of such events and no increased risk of medically-attended events in pregnant women. Screening of the Vaccine Adverse Event Reporting System in the United States has also indicated there are no differences in pregnancy complications or fetal outcomes, including spontaneous abortion, in pregnant women who receive TIV [17]. However, the majority of vaccine safety studies in pregnant women, including our own, have been observational in nature [18].

To our knowledge, this is the first study to directly compare the reactogenicity of influenza vaccine in pregnant women to a sample of non-pregnant women. It is interesting to note the higher incidence of fever observed in non-pregnant female HCWs, which may suggest a protective effect of pregnancy against febrile events. Such an occurrence is not implausible considering previous research has shown pregnancy can have a protective effect against medical conditions, such as breast cancer [19,20] and rheumatoid arthritis, due to hormonal and immunological changes induced by pregnancy [21]. Alternatively, it is possible these differences were observed due to reporting differences in the groups of women. Non-pregnant female HCWs are likely not a perfect comparison group. Because of their profession and potential knowledge of simple remedies, HCWs are a unique subset of vaccinees with distinctive health-seeking behaviours and perceptions of health. As a result, it is possible that the incidence of reported fever is more a reflection on the perception of fever, and the threshold for subjective fever may differ between HCWs and other cohorts. Another possible explanation for the observation that more HCWs reported fever is that pregnant women may expect or be accustomed to fluctuations in symptomatology related to their pregnancy and therefore do not attribute such symptoms to vaccination. However, these explanations are speculative, and additional studies would be required to explore further. In any event, our study found nothing to suggest pregnant women are more likely to report experiencing a reaction to inactivated influenza vaccine, compared to non-pregnant women of similar age.

There are several other limitations which should be considered when evaluating these results. First, the reactions were self-reported and generally not medically attended, thus they are subject to reporting biases. Secondly, we also identified demographic differences between these groups of women, most likely due to the younger age distribution of pregnant women compared to non-pregnant women and the preferential distribution of intradermal TIV in metropolitan HCW vaccination programs. However, we addressed these differences by controlling for differences in age and residence in the analysis.

## Conclusions

Our results indicate that pregnant women experience similar rates of vaccine-associated side effects as non-pregnant women, and these findings can be used to reassure pregnant women who are wary of influenza vaccination due to concerns about side effects. Continued monitoring of vaccine safety and reactogenicity is an integral component of vaccination campaigns [22]. Rapid, timely and relevant vaccine safety information can be collected using systems such as FASTMum. Integration of such data collection into vaccination programs would facilitate communication of vaccine safety information in a timely manner to pregnant women and antenatal care providers, promoting better informed decision-making regarding antenatal vaccination.
